# Surveillance for Waterborne Disease Outbreaks Associated with Drinking Water and Other Nonrecreational Water — United States, 2009–2010

**Published:** 2013-09-06

Despite advances in water management and sanitation, waterborne disease outbreaks continue to occur in the United States. CDC collects data on waterborne disease outbreaks submitted from all states and territories* through the Waterborne Disease and Outbreak Surveillance System.† During 2009–2010, the most recent years for which finalized data are available, 33 drinking water–associated outbreaks were reported, comprising 1,040 cases of illness, 85 hospitalizations, and nine deaths. *Legionella* accounted for 58% of outbreaks and 7% of illnesses, and *Campylobacter* accounted for 12% of outbreaks and 78% of illnesses. The most commonly identified outbreak deficiencies§ in drinking water-associated outbreaks were *Legionella* in plumbing¶ systems (57.6%), untreated ground water (24.2%), and distribution system deficiencies (12.1%), suggesting that efforts to identify and correct these deficiencies could prevent many outbreaks and illnesses associated with drinking water. In addition to the drinking water outbreaks, 12 outbreaks associated with other nonrecreational water** were reported, comprising 234 cases of illness, 51 hospitalizations, and six deaths. *Legionella* accounted for 58% of these outbreaks, 42% of illnesses, 96% of hospitalizations, and all deaths. Public health, regulatory, and industry professionals can use this information to target prevention efforts against pathogens, infrastructure problems, and water sources associated with waterborne disease outbreaks.

This report includes drinking water–associated outbreaks and other, nonrecreational waterborne disease outbreaks, in which the first illness occurred in 2009 or 2010. Outbreaks were reported to the Waterborne Disease and Outbreak Surveillance System through the electronic National Outbreak Reporting System†† as of October 3, 2012. Two criteria must be met for an event to be defined as a waterborne disease outbreak: 1) two or more persons must be linked epidemiologically by time, location of water exposure, and illness characteristics; and 2) the epidemiologic evidence must implicate water as the probable source of illness. Data requested for each outbreak include 1) the number of illnesses, hospitalizations, and deaths; 2) the etiologic agent (confirmed or suspected); 3) the implicated water system; 4) deficiencies contributing to the outbreak; and 5) the setting of exposure.

During 2009–2019, public health officials from 17 states reported 33 drinking water outbreaks (Table 1). The outbreaks resulted in 1,040 illnesses, 85 hospitalizations (8.2% of cases), and nine deaths. At least one etiologic agent was identified in all but one drinking water outbreak; *Legionella* was implicated in 19 outbreaks, 72 illnesses, 58 hospitalizations, and eight deaths, and *Campylobacter* was implicated in four single-etiology outbreaks involving 812 illnesses, 17 hospitalizations, and no deaths, as well as two multiple-etiology outbreaks resulting in 17 illnesses. The number and etiologies of drinking water outbreaks reported every year since 1971 were considered for comparison (Figure).

The etiologies, water systems, water sources, illnesses, and deficiencies identified for drinking water outbreaks and outbreak-associated cases were ranked in order of frequency (Table 2). *Legionella* caused the majority of outbreaks (57.6%); whereas non-*Legionella* bacteria caused the majority of illnesses (81.8%). The majority of outbreaks (75.8%) and outbreak-associated illnesses (79.4%) were linked to community water systems.§§ The majority of outbreaks (51.5%) and most illnesses (97.3%) occurred in systems that used ground water sources. The majority of outbreaks (57.6%) involved acute respiratory illness, whereas most outbreak-associated illnesses were acute gastrointestinal illness (92.6%). By deficiency categories, *Legionella* spp. in plumbing systems was present in the majority of outbreaks (19 [57.6%]); in three *Legionella* outbreaks, additional deficiencies in building-specific water treatment or plumbing systems were noted. Untreated ground water deficiency (i.e., contamination of ground water at the source) was identified in eight (24.2%) outbreaks, distribution system deficiency alone was identified in four (12.1%) outbreaks, and both deficiencies were identified in one outbreak (3.0%). Together, distribution system and untreated ground water deficiencies accounted for 965 (92.8%) of all outbreak-associated illnesses. All five outbreaks assigned a distribution system deficiency (i.e., distribution system or untreated ground water and distribution system) occurred in systems using ground water or mixed ground and surface water supplies; of these, three occurred in systems supplying unchlorinated ground water. Two of the distribution system-associated outbreaks (one in an unchlorinated supply) resulted from cross-connections (i.e., direct connections between piped water systems containing potable and nonpotable water).

In addition to the drinking water outbreaks, public health officials from 11 states reported 12 outbreaks associated with other nonrecreational water exposure (Table 1). The outbreaks included seven outbreaks of *Legionella* spp. resulting in 99 illnesses and six deaths. The water sources and settings for these outbreaks included cooling towers at a military facility and a hotel/motel setting, a mist/steam device in an industrial facility, an ornamental fountain in a health-care facility, and unidentified water exposures in long-term care, assisted-living, or rehabilitation facilities. The remaining outbreaks involved *Campylobacter* (two), *Giardia* (two), and acute gastrointestinal illness of unknown etiology (one) from ingesting water in various outdoor settings.

## Reported by

*Elizabeth D. Hilborn, DVM, Timothy J. Wade, PhD, Environmental Protection Agency. Lauri Hicks, DO, Laurel Garrison, MPH, Div of Bacterial Diseases, National Center for Immunization and Respiratory Diseases; Joe Carpenter, MS, Div of Healthcare Quality Promotion; Elizabeth Adam, MPH, Bonnie Mull, MPH, Jonathan Yoder, MPH, Virginia Roberts, MSPH, Julia W. Gargano, PhD, Div of Foodborne, Waterborne, and Environmental Diseases, National Center for Emerging and Zoonotic Infectious Diseases, CDC.* ***Corresponding contributor:*** *Julia W. Gargano, jgargano@cdc.gov, 404-718-4893.*

## Editorial Note

Since the early 20th century, water treatment processes and regulations have vastly reduced the transmission of illnesses through public drinking water supplies in the United States (1). The outbreaks reported during this surveillance period highlight several emerging and persisting public health challenges associated with drinking water systems. First, *Legionella* is the most frequently reported etiology among drinking water and other nonrecreational outbreaks. Fourteen of the 15 deaths reported were caused by *Legionella*, underscoring the need for improved *Legionella* control and mitigation methods. Second, the large proportion of outbreaks associated with untreated ground water (e.g., well water) indicates that additional efforts are needed to monitor ground water sources and protect them from contamination and to ensure that adequate, continuous disinfection is used when indicated by the results of monitoring and risk analyses (2). Finally, the large proportion (78%) of illnesses observed in outbreaks involving distribution system deficiencies emphasizes the importance of protecting, maintaining, and improving the public drinking water distribution system infrastructure (3) because these deficiencies can lead to widespread illness.

The total number of drinking water outbreaks reported during 2009–2010 (33) is similar to the number in previous 2-year intervals (e.g., 36 outbreaks during 2007–2008) (4). Although *Legionella* historically has been the most frequently reported etiology among drinking water outbreaks, during 2009–2010 *Legionella* comprised over half of reported drinking water outbreaks for the first time. In addition, *Legionella* also caused the majority of other nonrecreational water outbreaks (seven of 12). *Legionella* outbreaks are particularly challenging to prevent and control, in part because the organism multiplies in plumbing systems within buildings, which usually fall outside of regulatory oversight (5,6). Two of the 19 reported *Legionella* outbreaks occurred at health-care facilities where treatment systems to control *Legionella* growth had been installed, underscoring the limited effectiveness of engineering controls in complex plumbing systems.

In contrast to the emerging issue of *Legionella*, the problem of untreated ground water deficiencies in public and individual water systems persists. Full implementation of the Ground Water Rule, a federal regulation that aims to provide increased protection against microbial pathogens in public water systems that use ground water sources, might reduce the number of ground water outbreaks in public systems (2). However, this regulation does not address private wells, which the Environmental Protection Agency lacks the authority to regulate, emphasizing the continued need for education and outreach to private well owners to prevent outbreaks (7,8).

What is already known on this topic?

Despite advances in water management and sanitation, waterborne disease outbreaks continue to occur in the United States. CDC collects data on waterborne disease outbreaks submitted from all states and territories through the Waterborne Disease and Outbreak Surveillance System.

What is added by this report?

During 2009–2010, a total of 33 drinking water–associated outbreaks were reported to CDC, resulting in 1,040 cases of illness, 85 hospitalizations, and nine deaths. *Legionella* accounted for 58% of outbreaks and 7% of illnesses, and *Campylobacter* accounted for 12% of outbreaks and 78% of illnesses. The most commonly identified outbreak deficiencies were *Legionella* in plumbing systems (57.6%), untreated ground water (24.2%), and distribution system deficiencies (12.1%).

What are the implications for public health practice?

Efforts to identify and correct the deficiencies implicated in drinking water–related outbreaks, particularly deficiencies in distribution systems and untreated ground water systems, could prevent many outbreaks and illnesses. Additional research is needed to understand the interventions that are most effective for controlling growth of *Legionella* and reducing outbreaks of legionellosis.

Distribution system deficiencies continue to be a major contributor to drinking water outbreaks and outbreak-associated illnesses. Three outbreaks occurred in systems supplying unchlorinated water; if a disinfectant residual had been present, pathogens introduced by the distribution system deficiency might have been inactivated before the water reached consumers. Two outbreaks resulted from cross-connections between potable and nonpotable water pipes. The piecemeal nature of some infrastructure development might contribute to the occurrence of these cross-connections, highlighting the importance of distribution system monitoring and adherence to guidelines for the prevention of backflow of nonpotable water into the potable water supply (5,9).

The findings in this report are subject to at least two limitations. First, detection, investigation, and reporting of outbreaks are incomplete, and the level of surveillance and reporting activity varies across states and localities. Linking illness to drinking water is inherently difficult through outbreak investigation methods (e.g., case-control and cohort studies) because most persons have daily exposure to tap water (10). Environmental investigations provide information on deficiencies that contribute to outbreaks and strengthen evidence implicating drinking water as a common source of infection; however, capacity to conduct these investigations and report the results also differs by state and locality, and might change over time. For these reasons, outbreak surveillance should not be used to estimate the total number of illnesses from waterborne disease because most cases of waterborne disease are believed to occur sporadically or as part of outbreaks that are never recognized. Second, changes in the surveillance system occurred during this cycle, namely implementation of electronic reporting of waterborne disease outbreaks and the assignment of multiple deficiency categories for *Legionella* outbreaks. These changes do not affect the internal validity of the data in this report but might limit the ability to interpret trends in the number of outbreaks and deficiencies across reporting periods.

As observed in recent years, the proportion of outbreaks in the federally regulated portions of public water systems has declined, although these still contribute the majority of outbreak-associated illnesses. Deficiencies at points not under the jurisdiction of water utilities (e.g., private wells and plumbing systems) continue to cause illness. In addition, challenges with aging water infrastructure are ongoing, and efforts to understand the number of illnesses associated with drinking water distribution system deficiencies are needed. Partnerships between state and local public health agencies, as well as cooperation and coordination among epidemiologists, laboratorians, and environmental health specialists within agencies, are needed to optimize investigation and reporting of waterborne disease outbreaks. Additional information about the waterborne disease outbreaks reported during 2009–2010 is available at http://www.cdc.gov/healthywater/surveillance/drinking-surveillance-reports.html.

## Figures and Tables

**FIGURE:**
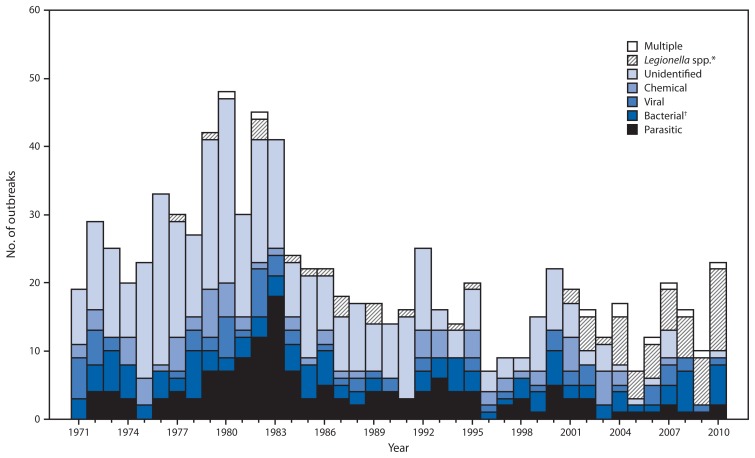
Number of waterborne disease outbreaks associated with drinking water (N = 851), by year and etiology — United States, 1971–2010 ^*^Legionnaires’ disease outbreaks were first reported to the Waterborne Disease and Outbreak Surveillance System in 2001; Legionnaires’ disease outbreaks before 2001 were added retrospectively during the 2007–2008 reporting period. ^†^Includes all bacteria except *Legionella*.

**TABLE 1:** Characteristics of waterborne disease outbreaks associated with drinking water (N = 33) and other nonrecreational water* (N = 12), by state/jurisdiction — Waterborne Disease and Outbreak Surveillance System, United States, 2009–2010

Exposure category and state/jurisdiction	Month	Year	Etiology	Predominant illness†	No. of cases	No. of hospitalizations§	No. of deaths¶	Water system**	Water source	Setting
**Drinking water**										
Florida	Jul	2009	*Legionella* sp.	ARI	2	2	0	Community	Well	Membership club
Idaho	May	2009	*Campylobacter* sp., *Giardia intestinalis*	AGI	7	0	0	Community	Well	Private residence
Maine	Jul	2009	Hepatitis A	Hep	2			Individual/Private	Well	Private residence
Maryland	Sep	2009	*Legionella pneumophila* serogroup 1, Knoxville 1	ARI	10	9	1	Community	Lake/Reservoir	Apartment/Condo
Nevada	Dec	2009	*Legionella pneumophila* serogroup 1	ARI	10	1	0	Community	Lake/Reservoir	Hotel/Motel
New York	Apr	2009	*Legionella pneumophila* serogroup 1	ARI	3	3	2	Community	Lake/Reservoir	Hospital/Health care
New York	Dec	2009	*Legionella pneumophila* serogroup 1	ARI	3	3	1	Community	Lake/Reservoir	Hospital/Health care
South Carolina	Jul	2009	*Legionella pneumophila* serogroup 1	ARI	3	3	0	Community	Ground water	Hotel/Motel
Utah	Jun	2009	*Legionella pneumophila* serogroup 1	ARI	5	5	0	Community	Well, spring	Hotel/Motel
Utah	Aug	2009	*Giardia intestinalis*	AGI	8	0	0	Community††	Well, surface water	Subdivision/Neighborhood
California	Jun	2010	Norovirus	AGI	47			Transient noncommunity	Well	Restaurant/Cafeteria
Georgia	Apr	2010	*Legionella pneumophila* serogroup 1	ARI	4	4	0	Community	Well, spring	Hotel/Motel
Illinois	Nov	2010	Unidentified§§	AGI; other¶¶	3	3	0	Commercially bottled	Unidentified	Church/Place of worship
Maryland	Aug	2010	*Legionella pneumophila* serogroup 1	ARI	2	2	0	Community	Surface water	Personal care home***
Minnesota	Jun	2010	*Giardia intestinalis*	AGI	6	0	0	Transient noncommunity	Well	State park
Missouri	Feb	2010	*Campylobacter jejuni*	AGI	16	5	0	Community	Well	Community/Municipality
Missouri	Mar	2010	*Campylobacter* sp.	AGI	67	4	0	Community	Well	Community/Municipality
Missouri	Apr	2010	*Escherichia coli* O157:H7	AGI	28	4	0	Community†††	Well	Membership club
Missouri	Nov	2010	*Escherichia coli* O157:H7	AGI	11	3	1	Individual/Private	Well	Private residence
Montana	Jul	2010	*Campylobacter jejuni*	AGI	101	6	0	Nontransient noncommunity	Well	Resort
Nevada	Dec	2010	*Legionella pneumophila* serogroup 1	ARI	4	2	1	Community	Well, river/stream	Hotel/Motel
New York	Apr	2010	*Legionella pneumophila* serogroup 1	ARI	3	3	1	Community	Lake/Reservoir	Hospital/Health care§§§
New York	Jun	2010	*Legionella pneumophila* serogroup 1	ARI	3	3	0	Community	Lake/Reservoir	Prison/Jail
New York	Jul	2010	*Legionella pneumophila* serogroup 1	ARI	2	2	0	Community	Lake/Reservoir	Hospital/Health care¶¶¶
New York	Jul	2010	*Legionella pneumophila* serogroup 1	ARI	5	3		Community	Lake/Reservoir	Hospital/Health care
Ohio	Feb	2010	*Legionella pneumophila*	ARI	3	3	0	Community	Unidentified	Long-term care facility
Pennsylvania	May	2010	*Legionella pneumophila* serogroup 1	ARI	3	3	1	Community****	Well	Personal care home
Pennsylvania	Jun	2010	*Legionella pneumophila* serogroup 1	ARI	2	2	0	Community	River/Stream	Apartment/Condo
Pennsylvania	Jul	2010	*Campylobacter jejuni*, *Cryptosporidium* sp.	AGI	10	0	0	Individual/Private	Well	Private residence
Utah	Apr	2010	*Campylobacter jejuni*	AGI	628	2	0	Community††	Well, spring	Community/Municipality
Utah	Aug	2010	*Legionella pneumophila* serogroup 1, Camperdown 1	ARI	2	2	1	Community	Spring, creek	Hotel/Motel
Utah	Dec	2010	*Legionella pneumophila* serogroup 1	ARI	3	3	0	Community	Well, surface water	Assisted living/Rehab
Vermont	Jan	2010	*Cryptosporidium* sp.	AGI	34	0	0	Individual/Private	Well	Vacation rental house
**Other nonrecreational water** *										
Alabama	Apr	2009	*Campylobacter jejuni*	AGI	11	0	0	Wilderness/Natural water source	River/Stream	Backcountry
Illinois	Sep	2009	*Legionella pneumophila* serogroup 1	ARI	8	8	2	Unknown	Ornamental fountain, spa, irrigation††††	Assisted living/Rehab
Missouri	Jul	2009	Unidentified	AGI	75	0	0	Wilderness/Natural water source	Spring	Camp/Cabin
New York	Aug	2009	*Giardia intestinalis*	AGI	26	1	0	Wilderness/Natural water source	Spring	Public outdoor area
Ohio	Sep	2009	*Legionella* sp.	ARI	2	2	0	Unknown	Unknown	Long-term care facility
Idaho	Jul	2010	*Campylobacter jejuni*	AGI	3	0	0	Wilderness/Natural water source	River/Stream	Backcountry
Michigan	Jul	2010	*Legionella pneumophila* serogroup 1	ARI	64	17	0	Cooling/Air conditioning	Cooling tower	Military facility
Mississippi	Jun	2010	*Legionella pneumophila* serogroup 1	ARI	9	6	1	Cooling/Air conditioning	Cooling tower	Hotel/Motel
Nevada	Jun	2010	*Giardia intestinalis*	AGI	20	1	0	Irrigation	Puddle/Canal/Swamp	Public outdoor area
New York	Nov	2010	*Legionella pneumophila* serogroup 1	ARI	4	4	0	Industrial/Occupational	Mist/Steam device	Factory/Industrial facility
Texas	May	2010	*Legionella pneumophila* serogroup 1	ARI	4	4	3	Unknown	Unknown	Long-term care facility
Wisconsin	Feb	2010	*Legionella pneumophila* serogroup 1	ARI	8	8	0	Ornamental	Ornamental fountain	Hospital/Health care

**Abbreviations:** AGI = acute gastrointestinal illness; ARI = acute respiratory illness; Hep = hepatitis; Other = undefined; illnesses, conditions, or symptoms that cannot be categorized as gastrointestinal, respiratory, ear-related, eye-related, skin-related, neurologic, hepatitis, or caused by leptospirosis.

* Nonrecreational category includes outbreaks involving water not intended for drinking and water of unknown intent but does not include recreational water exposures, which are reported separately.

† The category of illness reported by ≥50% of ill respondents. All legionellosis outbreaks were categorized as ARI.

§ Value was set to missing in reports where zero hospitalizations were reported and the number of persons for whom information was available also was zero.

¶ Value was set to missing in reports where zero deaths were reported and the number of persons for whom information was available also was zero.

** Community and noncommunity water systems are public water systems that have ≥15 service connections or serve an average of ≥25 residents for ≥60 days a year. A community water system serves year-round residents of a community, subdivision, or mobile home park. A noncommunity water system serves an institution, industry, camp, park, hotel, or business and can be nontransient or transient. Nontransient systems serve ≥25 of the same persons for >6 months of the year but not year-round (e.g., factories and schools), whereas transient systems provide water to places in which persons do not remain for long periods of time (e.g., restaurants, highway rest stations, and parks). Individual water systems are small systems not owned or operated by a water utility that have <15 connections or serve <25 persons.

†† A cross-connection between potable and nonpotable water sources resulting in backflow was a suspected or confirmed factor in this outbreak.

§§ Etiology unidentified: contamination of water with sodium hydroxide suspected based upon incubation period, symptoms, outbreak investigation, and laboratory findings.

¶¶ The other symptoms reported were chemical esophagitis and burns in mouth.

*** Facility had an onsite disinfection system that was not operational at the time of the outbreak.

††† Setting was a recreational facility with multiple buildings. A private well that was originally used for a residence was reclassified as a community water system as a result of the outbreak investigation.

§§§ The facility had an onsite chlorine dioxide system; however, there were indicators that the system was not being monitored properly at the time of the outbreak.

¶¶¶ The facility had an onsite chlorine dioxide system and was being monitored for *Legionella.*

**** Reported contributing factors included a temporary disruption in disinfection and a cross-connection between potable and nonpotable water sources resulting in backflow.

†††† Multiple water sources within the facility were identified as possible exposures in this outbreak.

**TABLE 2:** Etiology, water system,* water source, predominant illness,† and deficiencies§ associated with drinking water outbreaks (N = 33) and outbreak-related cases (N = 1,040), ranked in order of frequency — Waterborne Disease and Outbreak Surveillance System, United States, 2009–2010

Characteristic	Rank	Outbreaks (N = 33)			Cases (N = 1,040)		
	
		Category	No.	(%)	Category	No.	(%)
**Etiology**							
	1	*Legionella*	19	(57.6)	Bacteria, non-*Legionella*	851	(81.8)
	2	Bacteria, non-*Legionella*	6	(18.2)	*Legionella*	72	(6.9)
	3	Parasites	3	(9.1)	Viruses	49	(4.7)
	4	Multiple¶	2	(6.1)	Parasites	48	(4.6)
	5	Viruses	2	(6.1)	Multiple¶	17	(1.6)
	6	Chemical**	1	(3.0)	Chemical**	3	(0.3)
**Water system** *							
	1	Community††	25	(75.8)	Community††	826	(79.4)
	2	Individual	4	(12.1)	Noncommunity	154	(14.8)
	3	Noncommunity	3	(9.1)	Individual	57	(5.5)
	4	Bottled	1	(3.0)	Bottled	3	(0.3)
**Water source**							
	1	Ground water††	17	(51.5)	Ground water††	974	(93.7)
	2	Surface water	10	(30.3)	Surface water	43	(4.1)
	3	Mixed§§	4	(12.1)	Mixed§§	17	(1.6)
	4	Unknown	2	(6.1)	Unknown	6	(0.6)
**Predominant illness** †							
	1	ARI	19	(57.6)	AGI	963	(92.6)
	2	AGI	12	(36.4)	ARI	72	(6.9)
	3	Multiple¶¶	1	(3.0)	Multiple¶¶	3	(0.3)
	4	Viral hepatitis***	1	(3.0)	Viral hepatitis***	2	(0.2)
**Deficiency** §							
	1	*Legionella* spp. in plumbing system††† §§§	19	(57.6)	Distribution system¶¶¶	710	(68.3)
	2	Untreated ground water****	8	(24.2)	Untreated ground water****	154	(14.8)
	3	Distribution system¶¶¶	4	(12.1)	Untreated ground water and distribution system††††	101	(9.7)
	4	Untreated ground water and distribution system††††	1	(3.0)	*Legionella* spp*.* in plumbing system††† §§§	72	(6.9)
	5	Point of use (bottled)§§§§	1	(3.0)	Point of use (bottled)§§§§	3	(0.3)

**Abbreviations:** AGI = acute gastrointestinal illness; ARI = acute respiratory illness.

* Public water systems include community and noncommunity water systems that have ≥15 service connections or serve an average of ≥26 residents for ≥60 days a year. A community water system serves year-round residents of a community, subdivision, or mobile home park. A noncommunity water system serves an institution, industry, camp, park, hotel, or business.

† The category of illness reported by ≥50% of ill respondents. All legionellosis outbreaks were categorized as ARI.

§ Outbreaks are assigned one or more deficiency classifications. Deficiency names have been shortened to fit. A full description of CDC deficiency classification is available at http://www.cdc.gov/healthywater/surveillance/deficiency-classification.html.

¶ Two outbreaks had multiple etiologic agent types. In one outbreak, the etiologies were *Camplyobacter* sp. (i.e., bacterium) and *Giardia intestinalis* (i.e., parasite). In a second outbreak, the etiologies were *Campylobacter jejuni* (i.e., bacterium) and *Cryptosporidium* sp. (i.e., parasite).

** Etiology unidentified: contamination of water with sodium hydroxide suspected based upon incubation period, symptoms, outbreak investigation, and laboratory findings.

†† Ten outbreaks (763 cases) were in community water systems that used a ground water source exclusively. Of these, three outbreaks (111 cases) were in systems that were documented as not treating the water with a disinfectant, five outbreaks (645 cases) were in systems that added chlorine as a disinfectant, and two outbreaks (seven cases) had no information on disinfection documented.

§§ Includes outbreaks with mixed water sources (i.e., ground water and surface water). Three legionellosis outbreaks were associated with mixed source community water systems. One giardiasis outbreak was associated with a mixed source community water system.

¶¶ Symptoms for one outbreak caused by suspected chemical ingestion were categorized as AGI and other. The other symptoms reported were chemical esophagitis and burns in mouth.

*** Hepatitis symptoms are categorized separately. One outbreak of viral hepatitis was caused by hepatitis A.

††† Deficiency 5A. Drinking water; contamination of water at points not under the jurisdiction of a water utility or at the point of use: *Legionella* spp. in water system, drinking water.

§§§ Multiple deficiencies were assigned to three *Legionella* outbreaks. In two outbreaks, which contributed five cases, there was a deficiency in building/home-specific water treatment. In one outbreak, which contributed three cases, there was a treatment deficiency outside of the building/home as well as a deficiency in the plumbing system.

¶¶¶ Deficiency 4. Drinking water; contamination of water at/in the water source, treatment facility, or distribution system: distribution system deficiency, including storage (e.g., cross-connection, backflow, and contamination of water mains during construction or repair). The four outbreaks involving distribution system deficiency included three outbreaks in systems using only ground water sources and one outbreak in a system using both ground and surface water. Two of the three ground water systems disinfected with chlorine, one ground water system and the system using ground and surface water did not disinfect.

**** Deficiency 2. Drinking water; contamination of water at/in the water source, treatment facility, or distribution system: untreated ground water.

†††† Outbreak involved both Deficiency 2 and Deficiency 4. Outbreak occurred in a nontransient, noncommunity water system using a ground water source that was not treated with a disinfectant.

§§§§ Deficiency 11C. Drinking water; contamination of water at points not under the jurisdiction of a water utility or at the point of use: contamination at point of use, commercially bottled water.

## Footnotes

* Outbreak reports can be submitted by public health agencies in U.S states, the District of Columbia, Guam, Puerto Rico, the Marshall Islands, the Federated States of Micronesia, the Commonwealth of the Northern Mariana Islands, Palau, and the U.S. Virgin Islands.

† A description of the Waterborne Disease and Outbreak Surveillance System is available at http://www.cdc.gov/healthywater/surveillance/index.html.

§ Outbreaks are assigned one or more deficiency classifications based on available data. The classifications provide information about how the water became contaminated, water system characteristics, and factors leading to waterborne disease outbreaks. A full description of CDC deficiency classification is available at http://www.cdc.gov/healthywater/surveillance/deficiency-classification.html.

¶ “Plumbing” refers to the pipes that are within a building or within a service line leading into a building, distinguished from the distribution system of pipes that comprise the water supply.

** Nonrecreational category includes outbreaks involving water not intended for drinking and water of unknown intended use, but does not include recreational water exposures, which are reported separately.

†† A description of the National Outbreak Reporting System is available at http://www.cdc.gov/nors/about.html. This reporting platform allows states to submit reports of waterborne, foodborne, animal-to-person, person-to-person, and unknown outbreaks to CDC. The Waterborne Disease and Outbreak Surveillance System transitioned from a paper-based system to electronic reporting through the National Outbreak Reporting System in 2009; this is the first report of data collected in the electronic system.

§§ Public water systems include community and noncommunity water systems, which have ≥15 service connections or serve an average of ≥26 residents for ≥60 days a year. A community water system serves year-round residents of a community, subdivision, or mobile home park. A noncommunity water system serves an institution, industry, camp, park, hotel, or business.
